# Maintaining and broadening DICOM adoption in digital pathology: A response to “wearing a fur coat in the summertime”

**DOI:** 10.1016/j.jpi.2025.100517

**Published:** 2025-09-13

**Authors:** Mustafa Yousif, Brian Napora, John Groth, Kenneth Philbrick, Norman Zerbe, David Clunie, Ulysses G.J. Balis

**Affiliations:** a University of Michigan School of Medicine, Michigan Medicine 1500 E. Medical Center Drive, Ann Arbor, MI 48109, USA.; b Gestalt Diagnostics, 850 E. Spokane Falls Blvd, Spokane, WA 99202, USA.; c Endeavor Health (NorthShore Evanston Hospital) 2650 Ridge Ave, Evanston, IL 60201, USA.; d Google Inc (now Google LLC) 1600 Amphitheatre Pkwy, Mountain View, CA 94043, USA.; e Charité Universitätsmedizin Berlin, Campus Charite Mitte Chariteplatz 1, 10117 Berlin, Germany.; f PixelMed Publishing, LLC, Bangor, PA, United States of America

## Members of the DICOM Working Group-26 (Pathology)

Mustaf Yousif, MD, Michigan Medicine

Brian Napora, Gestalt Diagnostics

Kenneth Philbrick, Ph.D., Google

John Groth, MD, Endeavor Health

David Clunie, Pixelmed Publishing

Ulysses Balis, MD, Michigan Medicine

Norman Zerbe, PhD

Steven Hart, Ph.D., Mayo Clinic

Eric Martin, Proscia

Terence Chiang, Song Yi System

Emilio Madrigal, DO, Massachusetts General Hospital

Chung-Yueh Lien, National Taipei University

Markus D. Hermann, College of American Pathologists

Marcial Garcia Rojo, Hospital Universitario de Jerez

Richard Preston, Hamamatsu Photonics Europe

Robin Breslin, IHE United Kingdom

Jörg Riesmeier, IT Consultant

Bruno Lafflin, AGFA HealthCare

Tamás Sárga, 3D Histech

Mohannad Hussain, Radical Imaging

Melanie Shedd, Voicebrook

Pau-Choo Chung, National Cheng Kung University

Michael Riben, MD., M.D. Anderson Cancer Center

Daniel Cohen, MD

Johan Doré, Visopharm

Ruben Fernandes, Sectra ImIT

Sandro Maenza, Siemens Healthineers

Ingo Wilkening, Siemens Healthineers

Antje Schroeder, Siemens Healthineers

Björn Nolte, Siemens Healthineers

Klaus Wolf, Siemens Healthineers

Daniela Schacherer

Alessandro Noriaki Ide, Siemens Healthineers

Fran Carrasco, Meditecs

Michael Knapp, J4care GmbH

Francesca Vanzo

Giovanni Lujan, MD, The Ohio State University College of Medicine

Dibson Dibe Gondim MD, University of Louisville

Jean-François Pomerol, Tribun Health

Luís Bastião Silva, BMD Software

Rushabh A. Mehta, identify.bio

Ty Usrey, Leica Biosystems

Keith J Kaplan MD., Versant Diagnostics

Charles Cheng, AIxMed

Sébastien Jodogne, Orthanc project

Melissa Alexander, MD, PhD, FCAP

Tom Kimpe, Barco NV

Abhishek Garhia, Pramana

John Memarian, CitiusTech

## DICOM Pathology Working Group-26

Dear Editors,

We write as members of DICOM Working Group 26 in response to Dr. Gershkovich's recent article, “Wearing a fur coat in the summertime: Should digital pathology redefine medical imaging?” While we appreciate his contributions, several assertions about the DICOM standard are incomplete or inaccurate, risking distraction from recent substantial advancements.

Reliable communication of medical images is important for the best possible patient care across worldwide medical practice. DICOM is the standard through which medical imaging is communicated. It defines representations for how pixel data are encoded in a variety of formats binary: JSON, XML, and other representations. DICOM also specifies the communication standards through which these representations can be searched, stored, indexed, and retrieved. In comparison, traditional image formats commonly define the representation of the pixel data but lack mechanisms that function to facilitate their communication.

Collaboration among standards groups, regulatory agencies, and vendors has made DICOM a valuable standard for the representation and communication of digital pathology images. Maintaining this unified approach is crucial for continued growth of digital pathology as a vital component of an integrated healthcare ecosystem. The maturity of this collaboration is evident in the current interoperability Connectathon sponsored by both the DICOM (WG-26) and IHE (PaLM) standards digital pathology working groups. In this Connectathon, over 30 digital pathology vendors and healthcare providers from around the world registered to demonstrate interoperability between LIS, WSI Scanners, Image Archives, Image Viewers, and pathology AI Creators. One exciting example of the integration achieved is actually seeing slides from nearly all the major scanner vendors being analyzed by AI algorithms from different vendors and all displayed in a single medical imaging viewer.

Interoperability of pathology medical images is also demonstrably operational. Medical facilities in Europe, Asia, and the Americas have all leveraged DICOM and other standards to reduce complexity and expedite deployment. One recent example is the University of Michigan Medicine's digital pathology deployment. At Michigan Medicine, the radiology and pathology departments share a common PACS using DICOM. The success of this commonality was recently showcased this past January in *CAP Today's* feature article, which quoted several of the authors of this letter (Balis and Yousif) concerning the reality that the previous concerns about storage, computation, and image quality with respect to digital pathology workflow have been resolved, with DICOM being a key enabling tool, and not an obstacle. Another current example is at Endeavor Health, where a DICOMweb and IHE-based digital pathology deployment plays a central role in the organization's enterprise imaging strategy. This implementation is actively demonstrating the benefits of metadata enrichment for clinical deployment and interoperability.

A subject of concern for Dr. Gershkovich centered upon the apparent reduplicative nature of metadata representation within the DICOM standard, over data that can be stored in other sectors of the health system. While the standard explicitly mandates only the fields essential for storing and decoding medical images, this incremental capacity allows for the inclusion of supplemental metadata, creating self-contained datasets. This is a highly useful feature of DICOM, as it allows for modular storage and co-location of critically important patient health information along with image data, enabling, for example, an intact diagnostic pathology workflow even when other supporting systems might be unavailable, such as in the case of a natural disaster or other technical interruption.

DICOM supports patient confidentiality through field-level pseudonymization and can work with modern encryption and access controls technologies that should be a part of any enterprise healthcare information security strategy.

Current momentum across the industry supports DICOM's adoption. FDA reviewers are familiar with the standard and recognize it as a voluntary consensus standard.^1^ Many vendors produce and consume native DICOM files, and LIS vendors embed metadata directly, reducing errors and improving safety.

HL7 FHIR and other standards related to the exchange of structured clinical information are vital for truly interoperable clinical workflows. In fact, FHIR has a dedicated Imaging Study resource that is designed to work with DICOM. However, for exchange of medical image data, DICOM remains unmatched because of its industry adoption, clinical acceptance, available tooling, existing IT infrastructure, and regulatory support. Moving away from this standard now would fragment efforts and delay progress.

While DICOM has proven to be a highly useful standard, we recognize that there is room for improvement to further promote its adoption in digital pathology. We encourage the pathology community to maintain focus on solidifying and broadening DICOM adoption through continued investment by vendors and increased demand from healthcare providers. We also invite those with meaningful suggestions for improving the standard to participate in the WG-26 monthly meetings. This joint effort will accelerate improvements in efficiency, accuracy, and patient safety, which are the goals of any truly interoperable healthcare IT system.

Thank you for facilitating this dialogue. We look forward to continued collaboration on a safe, unified, open set of standards for digital pathology.

Sincerely,

DICOM Working Group-26 (Pathology) – Endorsing Members

• Abhishek Garhia, Pramana
• Alessandro Noriaki Ide, Siemens Healthineers
• Bjorn Nolte, Siemens Healthineers
• Bruno Lafflin, AGFA HealthCare
• Charles Cheng, AIxMed
• Chung-Yueh Lien, National Taipei University
• Daniel Cohen, MD
• Daniela Schacherer
• David Clunie, Pixelmed Publishing
• Dibson Dibe Gondim, MD, University of Louisville
• Emilio Madrigal, DO, Massachusetts General Hospital
• Eric Martin, Proscia
• Fran Carrasco, Meditecs
• Francesca Vanzo
• Giovanni Lujan, MD, The Ohio State University College of Medicine
• Jean-Francois Pomerol, Tribun Health
• Johan Dore, Visopharm
• John Groth, MD, Endeavor Health
• John Memarian, CitiusTech
• Jorg Riesmeier, IT Consultant
• Keith J. Kaplan, MD, Versant Diagnostics
• Klaus Wolf, Siemens Healthineers
• Markus D. Hermann, College of American Pathologists
• Marcial Garcia Rojo, Hospital Universitario de Jerez
• Melissa Alexander, MD, PhD, FCAP
• Melanie Shedd, Voicebrook
• Michael Knapp, J4care GmbH
• Michael Riben, MD, M.D. Anderson Cancer Center
• Mohannad Hussain, Radical Imaging
• Mustaf Yousif, MD, Michigan Medicine
• Norman Zerbe, PhD
• Pau-Choo Chung, National Cheng Kung University
• Richard Preston, Hamamatsu Photonics Europe
• Robin Breslin, IHE United Kingdom
• Ruben Fernandes, Sectra ImIT
• Rushabh A. Mehta, identify.bio
• Sandro Maenza, Siemens Healthineers
• Sebastien Jodogne, Orthanc Project
• Steven Hart, PhD, Mayo Clinic
• Tamas Sarga, 3D Histech
• Terence Chiang, Song Yi System
• Tom Kimpe, Barco NV
• Ty Usrey, Leica Biosystems
• Ulysses Balis, MD, Michigan Medicine

## Declaration of competing interest

The authors declare that they have no known competing financial interests or personal relationships that could have appeared to influence the work reported in this paper.

## Footnotes

1 httpsn//www.accessdata.fda.gov/scripts/cdrh/cfdocs/cfStandards/detail.cfm?standard__identification_no=45752.

